# Experimental and thermodynamic modeling of sumatriptan solubility in supercritical carbon dioxide for green pharmaceutical applications

**DOI:** 10.1038/s41598-025-20442-7

**Published:** 2025-10-21

**Authors:** Saud Bawazeer

**Affiliations:** Department of Pharmaceutical Science, College of Pharmacy, Umm Al-Qura University, Makkah, Saudi Arabia

**Keywords:** Solubility, Sumatriptan, Supercritical carbon dioxide, Modeling, Chemistry, Environmental sciences

## Abstract

This study examined the solubility of sumatriptan when dissolved in supercritical carbon dioxide. The temperature of the system was varied between 308 and 338 K, and the pressure was ranged between 10 and 30 MPa. The experimental mole fraction and the solubility ranged from 4.30 × 10^−6^ to 5.26 × 10^−5^ and from 0.010 to 0.278 g/l respectively with a cross over point of 15 MPa. A total of four semi-empirical models (Chrastil, Bartle et al., K-J, and MST) were evaluated for their potential in facilitating both the correlation and the self-consistency of the data. The K-J method yielded the best outcome, with an AARD of 8.21% and a R_adj_ of 0.991. These models also helped estimate thermal enthalpies (${\Delta H}_{t}$ = 40.96 kJ mol^−1^, ${\Delta H}_{vap}$ = 59.83 kJ mol^−1^, and ${\Delta H}_{sol}$ = − 18.87 kJ mol^−1^). Thermodynamic modeling using the PC-SAFT, Peng-Robinson and Soave–Redlich–Kwong combined with mixing rules showed that the PC-SAFT offered better accuracy (AARD = 11.75%, R_adj_ = 0.988). These findings provide essential experimental data and validated predictive models that are critical for designing eco-friendly supercritical fluid processes, such as the supercritical anti-solvent.

**Supplementary Information:**

The online version contains supplementary material available at 10.1038/s41598-025-20442-7.

## Introduction

Sumatriptan is one of the prescribed medications for the acute treatment of migraine attacks. It is a serotonin receptor agonist that targets the 5-HT1B/1D receptors. Migraines are debilitating neurological disorders characterized by intense headaches accompanied by symptoms such as nausea, light sensitivity, and aura. Despite its clinical success and widespread use, sumatriptan’s therapeutic efficacy is significantly hindered by its low oral bioavailability, mainly attribute to extensive first-pass metabolism in the liver^1–4^. Although sumatriptan is the most common and preferred form of drug delivery, its oral bioavailability is only about 14%. This is in stark contrast to subcutaneous administration, where bioavailability reaches 96%. This emphasizes the challenges of achieving optimal systemic levels of the drug through oral formulations. The low oral bioavailability stems from poor absorption and rapid hepatic clearance, which limits drug plasma concentrations and reduces its clinical effectiveness in quickly alleviating migraine symptoms^3–5^. Given these limitations, researchers and pharmaceutical developers have explored strategies to enhance sumatriptan bioavailability and overcome the drawbacks of oral administration. One promising approach is the development of sublingual films: thin, fast-dissolving strips that release the drug rapidly under the tongue. One important factor that influences sumatriptan’s formulation and absorption is its inherent water solubility. Although quantitative measurements of its aqueous solubility are scarce, various pharmaceutical techniques aim to address solubility challenges. For instance, the preparation of solid and nano-dispersions has been demonstrated to significantly enhance the dissolution rate of poorly soluble drugs. These formulations have been demonstrated to enhance surface area for dissolution, thereby optimizing bioavailability and therapeutic efficacy^1,6,7^.

Particle engineering is crucial for enhancing drug solubility and absorption. The surface area and morphology of active pharmaceutical ingredients (APIs) directly impact dissolution rates, which are key determinants of bioavailability. However, conventional methods for reducing particle size, such as milling and solvent-based recrystallization, often introduce issues like broad particle size distributions, potential polymorphic transformations that may affect drug stability, and contamination from residual solvents^8–10^. Because of this, supercritical fluid (SCF) is seen as a good option for the pharmaceutical industry. It can be used to make medicines in a way that is good for the environment. It is also precise and can be used to make a lot of medicines. Supercritical carbon dioxide (SC-CO_2_) is a popular choice because it has many advantages. It is non-toxic, non-flammable, inexpensive, and easy to get. The temperature (304.1 K) and pressure (73.8 bar) are mild, which makes it compatible with thermally sensitive compounds like sumatriptan^11–15^. The variable solvent power of SC-CO_2_ enables precise control of crystallization and particle formation via several techniques that were investigated by many researchers^16–18^. In addition, It emphasizes the potential of SC-CO_2_ as an environmentally friendly media for drug formulation, nanoparticle production, and enhanced drug delivery systems.

Production sumatriptan’s particle size and morphology through SCF based methods could significantly improve its efficacy by enhancing dissolution kinetics and absorption rates. However, the lack of fundamental data on sumatriptan’s solubility in SC-CO_2_ currently limits the rational design and industrial scaling of these processes. Comprehensive solubility measurements over relevant pressure and temperature ranges are essential to understanding solute–solvent interactions and enabling process optimization. This study aims to address this critical knowledge gap by determining the solubility of sumatriptan in SC-CO_2_ under conditions applicable to pharmaceutical manufacturing. In addition to the experimental investigations, an attempt was made to correlate the experimental data using four density-based models like Chrastil, Bartle, et al., K-J, and MST. Three thermodynamically rigorous equations of state, PC-SAFT, Peng-Robinson and Soave–Redlich–Kwong, were applied with suitable mixing rules to model phase behavior and provide deeper insight into the solute–solvent system. The model performance was assessed using statistical metrics, including AARD and the coefficient of determination (adjusted) (R_adj_), to identify the most accurate approach for predicting sumatriptan solubility in SC-CO_2_.

## Materials and methods

### Materials

In this study sumatriptan with CAS number 103628-46-2 was purchased from Sigma Aldrich compony. The details were reported in Table 1.

**Table 1:** The properties of sumatriptan.

Compound	Formula	Structure	M_W_	CAS number
Sumatriptan	C_14_H_21_N_3_O_2_S		295.40	103628-46-2

## Methods

### The solubility measurement

Detailed procedure for measuring the solubility of sumatriptan using gravimetric techniques was explained as follows:

#### Prepare the high-pressure system

**Fig. 1:**
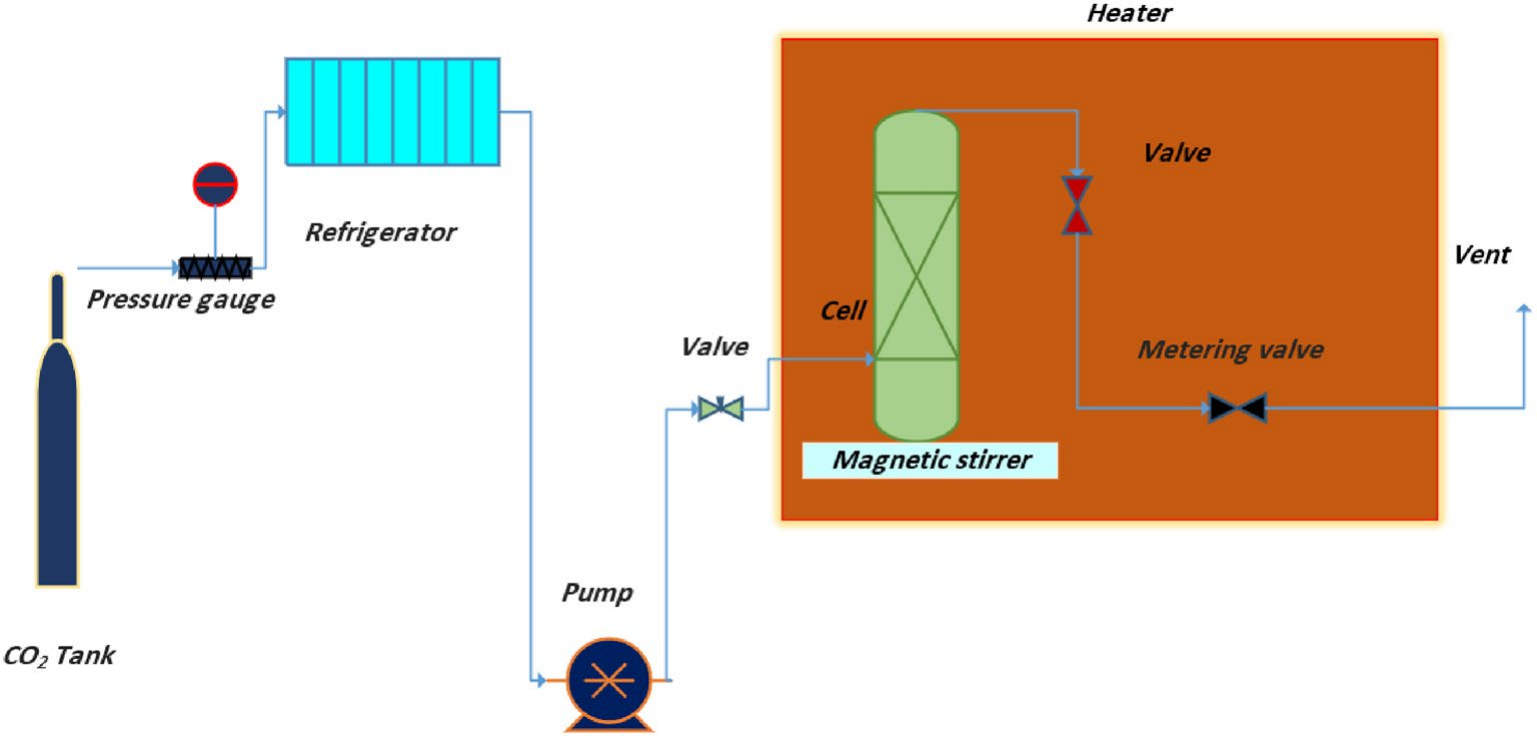
High pressure system designed for measuring the solubility.

Use a specialized high-pressure system designed for solubility measurements, as shown in Fig. 1.

The system should include a cylindrical vessel with an internal volume of 200 mL. The vessel was made of materials that can withstand high pressures (up to 40 MPa) and high temperatures (up to 423 K). Verify that all seals, valves, and fittings are properly installed and leak-free to maintain system integrity under high-pressure conditions. Calibrate the system’s pressure and temperature sensors to ensure accurate readings throughout the experiment.

#### Prepare sumatriptan tablets

Using an analytical balance with a sensitivity of 0.01 mg, weigh exactly 2000 mg of sumatriptan powder to ensure precise measurements. Use a tablet press to compact the powder into tablets with a uniform diameter of approximately 5 mm to ensure consistent volume. Inspect the tablets for structural integrity to prevent disintegration during the experiment. Place the tablets carefully in a basket inside the equilibrium vessel to maximize their exposure to SC-CO_2_.

#### Introduce carbon dioxide

Connect a high-precision pump to the vessel to deliver CO_2_ in a controlled manner. Gradually introduce CO_2_ into the vessel, increasing the pressure by 0.1 MPa increments to avoid sudden pressure surges that could affect system stability. Monitor the pressure using the integrated digital pressure gauge. Once the target pressure is achieved, adjust the pump to maintain a stable CO_2_ flow and consistent pressure within ± 0.01 MPa.

#### Set and control experimental conditions

Configure the system to operate under temperature and pressure conditions that are suitable for SC-CO_2_. Typically, these conditions fall within the range of 308–423 K and 10–40 MPa, depending on the experimental design. Use a high-precision thermocouple to monitor and maintain the temperature within ± 0.1 K to ensure thermal stability throughout the experiment. Activate the stirring mechanism and set the speed to 250 rpm to promote uniform mixing of the SC-CO_2_ and the tablets and facilitate consistent dissolution.

#### Achieve equilibrium

Run the system with continuous stirring at 250 rpm for 300 min to allow the drug to reach solubility equilibrium with the SC-CO_2_. Periodically monitor the system’s pressure and temperature to ensure they remain stable within the specified ranges.

#### Depressurize the system

After the 300 min equilibration period, rapidly depressurize the vessel to ambient conditions to halt the dissolution process. Depressurize the system in a controlled manner to loss of the sample.

#### Measure the undissolved drug

Carefully remove the basket containing the remaining drug tablets from the vessel. Gently clean the tablets to remove any CO_2_. If necessary, dry the tablets under controlled conditions (e.g., in a desiccator) to remove adsorbed CO_2_. Weigh the undissolved drug using an analytical balance with a sensitivity of 0.01 mg. Perform replicate experiments (e.g., at least three runs) to ensure reproducibility. Then, calculate the average solubility and standard deviation.

#### Calculate solubility

Use the following formula to calculate the mass of dissolved sumatriptan:

1 $${\text{m}}_{\text{dissolved}}={\text{m}}_{\text{initial}}-{\text{m}}_{\text{undissolved }}$$

Include the molecular weights of sumatriptan (295.4 g/mol) and CO_2_ (44.01 g/mol) in the mole fraction calculations.

2 $$\text{Mole of drug}=\frac{{\text{m}}_{\text{dissolved}}}{{\text{M}}_{\text{w},\text{ sumatriptan}}}$$

3 $$\text{Mole of }{\text{CO}}_{2}=\frac{{\text{m}}_{{\text{CO}}_{2}}}{{\text{M}}_{\text{w}, {\text{ CO}}_{2}}}$$

Determine the mole fraction of dissolved drug in SC-CO_2_ by dividing the moles of the dissolved drug by the total moles in the system (drug + CO_2_).

4 $$\text{y}=\frac{\text{Mole of sumatriptan }}{\left(\text{Mole of sumatriptan }+\text{Mole }{\text{of CO}}_{2}\right)}$$

If necessary, the solubility (mg/L) can be calculated by incorporating the density of SC-CO_2_ at the experimental temperature and pressure.

5 $$Solubility =\frac{\rho \times {M}_{sumatriptan} \times y}{{M}_{C{O}_{2}}\times \left(1-y\right)}$$

## Modeling

### Density based models

Various density-based models have been used to study the solubility of sumatriptan in SC-CO_2_. The models used in this study were developed by Chrastil^19^, Bartle et al.^20^, MST^21^, and K-J^22^. The equations for these models are presented in Table 2, and further details can be found in the referenced literature.

**Table 2:** The list of density-based models.

Binary system		
Model	Equation	Ref
Chrastil	${\text{lny}}_{2}={a}_{0}+{a}_{1}ln{\rho }_{1}+\frac{{a}_{2}}{T}$	^19^
Bartle et al	$ln\left(\frac{{y}_{2}P}{{P}_{ref}}\right)={a}_{0}+\frac{{a}_{1}}{T}+{a}_{2}\left({\rho }_{1}-{\rho }_{ref}\right)$	^20^
MST	$Tln\left({y}_{2}P\right)={a}_{0}+{a}_{1}{\rho }_{1}+{a}_{2}T$	^23^
Kumar and Johnston (KJ)	$ln{y}_{2}={a}_{0}+{a}_{1}{\rho }_{1}+\frac{{a}_{2}}{T}$	^22^

The Chrastil model posits that a solute molecule binds with *k* molecules of the supercritical solvent to form an equilibrium solvate complex described by a three-parameter equation. The total heat, $\Delta {H}_{t}$, is calculated from the third parameter (${a}_{2}=\frac{\Delta {H}_{total}}{R}$).

In the Bartle model, a_2_ determines the solute’s heat of vaporization $\Delta {H}_{vap}$. Integrating $\Delta {H}_{t}$ from the Chrastil model and $\Delta {H}_{vap}$ yields an estimate of the heat of solvation $\Delta {H}_{sol}$ for a solute-CO_2_ system.

The MST^21^ is based on a Helmholtz energy expansion near the solvent’s critical point and captures mixture behavior at infinite dilution. Due to the lack of sublimation vapor pressure data, the model incorporates a Clausius-Clapeyron expression, yielding a three-parameter model for solid solubility.

The K-J model^22^ has three adjustable variables and takes into account temperature, and solvent density. It demonstrates strong alignment with experimental solubility data, which is an important consideration in the field.

### Equation of state (EoS)

In the context of modeling gas–solid equilibrium, it is imperative that the temperature, pressure, and fugacity of the two phases are equal to ensure the stability of the equilibrium. Equations of state are instrumental in determining the fugacity coefficients of components in SC-CO_2_ to facilitate solubility predictions.

The general fugacity equality can be expressed as follows:

6 $${f}_{2}^{SC-C{O}_{2}}={f}_{2}^{solid}$$

The mole fraction (${y}_{2}$) of a compound in SC-CO_2_ was calculated using the following equation:

7 $${y}_{2}=\frac{{P}_{2}^{sub}\left(T\right)}{P}\frac{{\varnothing }_{2}^{sat}\left(T\right)}{{\varnothing }_{2}\left(T,P,y\right)}exp\left[\frac{{v}_{2}^{s}\left(P-{P}_{2}^{sub}\left(T\right)\right)}{RT}\right]$$

Fugacity coefficients ${\varnothing }_{i}$ ​ are calculated using the chosen Equation of State.

8 $$RTln {\varnothing }_{i}=-RTlnZ+{\int }_{V}^{\infty }\left[{\left(\frac{\partial P}{\partial {n}_{i}}\right)}_{T,V,{n}_{j}\ne {n}_{i}}-\frac{RT}{V}\right]dV$$

In this framework, the solute fugacity coefficient, designated hereinafter as ${\varphi }_{s}$, indicates the sublimation pressure of the APIs, which is typically low. Therefore, it can be deduced that ${\varphi }_{s}$ is equivalent to one. To ascertain the value of ${\varphi }_{s}^{sat}$, it is imperative to employ the appropriate model to represent a process that is in equilibrium. The EoSs were then evaluated using the SRK, PR, and PC-SAFT models.

### Peng-Robinson

The Peng-Robinson is a cubic EoS for supercritical fluids and gas mixtures. It expresses pressure as:

9 $$P=\frac{RT}{v-b}-\frac{a\left(T\right)}{v\left(v+b\right)+b\left(v-b\right)}$$

10 $$a=0.45724\frac{{R}^{2}{T}_{c}^{2}}{{P}_{c}}\alpha \left({T}_{r},\omega \right)$$

11 $$b=0.0778\frac{R{T}_{c}}{{P}_{c}}$$

12 $$\alpha \left({T}_{r},\omega \right)={\left[1+k(1-\sqrt{{T}_{r})}\right]}^{2}$$

13 $$k=0.37464+1.5422\omega -0.26992{\omega }^{2}$$

### SRK (Soave–Redlich–Kwong equation)

The SRK is another cubic equation used for thermodynamic modeling of supercritical fluids:

14 $$P = \frac{RT}{{\nu - b}} - \frac{a(T)}{{\nu \,\,(\nu + b)}}$$

where,

15 $$a(T) = \frac{{0.42747R^{2} T_{c}^{2} }}{{P_{c} }}\, \times \,\underbrace {{(1 + m(1 - T_{r}^{0.5} ))^{2} }}_{{\alpha (T_{r,\omega } )}}\,\,\,\,\,\,\,\,\,\,\,{\text{and}}\,\,\,\,\,\,m = 0.480 + 1.574\,\omega - 0.176\,\omega^{2}$$

16 $$b = \frac{{0.08664RT_{c} }}{{P_{c} }}$$

The mixing rule of were defined as^24^:

17 $${a}_{m}=\sum_{i}\sum_{j}{x}_{i}{x}_{j}{a}_{ij}$$

18 $${b}_{m}=\sum_{i}\sum_{j}{x}_{i}{x}_{j}{b}_{ij}$$

Here, ${a}_{ij}$ and ${b}_{ij}$ were calculated as follows^24^:

19 $${a}_{ij}={\left({a}_{i}{a}_{j}\right)}^{0.5}\left(1-{k}_{ij}\right)$$

20 $${b}_{ij}=\left(\frac{{b}_{i}+{b}_{j}}{2}\right)\left(1-{l}_{ij}\right)$$

#### PC-SAFT

SAFT equations are considered a unique category of models used to establish correlations between the solubility of substances in SC-CO_2_. A comparison of SAFT equations with other thermodynamic models reveals that the former has a more complex structure. Notably, the general and partial forms of these models have been used in many research studies^25–32^. The utilization of these models is contingent upon a comprehensive understanding of the molecular relationship between carbon dioxide and the solutes. A substantial corpus of research has identified these parameters as being subject to modification. The PC-SAFT model is predicated on the residual molar Helmholtz energy (${\widetilde{\text{a}}}^{\text{res}}$), incorporating the $\text{and }$ which constitute the primary foundation of the model in this study^33^:

21 $${\tilde{\text{a}}}^{{{\text{res}}}} = {\text{A}}/{\text{NKT}} = a^{hc} + a^{disp}$$

The term "${a}^{hc}$" is defined as follows, according to the principles of first-order thermodynamic perturbation theory^28^:

22 $$\tilde{a}^{hc} = (\mathop \sum \limits_{i = 1}^{N} y_{i} m_{i} )\tilde{a}^{hs} - \mathop \sum \limits_{i = 1}^{N} y_{i} \left( {m_{i} - 1} \right)lng_{ii}^{hs} \,\sigma_{ii}$$

As well as, the ${a}^{disp}$ is obtained by the below equation^28^:

23 $$\begin{gathered} \tilde{a}^{disp} = - 2\pi \underbrace {{\frac{6}{\pi }\eta \left( {\mathop \sum \limits_{i = 1}^{N} y_{i} m_{i} d_{i}^{3} } \right)^{ - 1} }}_{\rho }\left[ {l_{1,xk} \overline{{m^{2} \varepsilon \sigma^{3} }} + l_{1} \overline{{\left( {m^{2} \varepsilon \sigma^{3} } \right)_{xk} }} } \right] - \pi \frac{6}{\pi }\eta \left( {\mathop \sum \limits_{i = 1}^{N} y_{i} m_{i} d_{i}^{3} } \right)^{ - 1} \hfill \\ \left\{ {\left[ {m_{k} C_{1} l_{2} + \overline{m}C_{1,xk} l_{2} + \overline{m}C_{1} l_{2,xk} } \right] \times \overline{{m^{2} \varepsilon^{2} \sigma^{3} }} + \overline{m}C_{1} l_{2} \overline{{\left( {m^{2} \varepsilon^{2} \sigma^{3} } \right)_{xk} }} } \right\} \hfill \\ \end{gathered}$$

The conventional combination rules are employed to ascertain the two cross parameters (${\upvarepsilon }_{\text{ij}}$ and ${\sigma }_{ij}$)^28^:

24 $${\upsigma }_{\text{ij}}=\frac{1}{2}\left({\upsigma }_{\text{i}}+{\upsigma }_{\text{j}}\right)$$

25 $${\upvarepsilon }_{\text{ij}}=\sqrt{{\upvarepsilon }_{\text{i}}{\upvarepsilon }_{\text{j}}}\left(1-{\text{k}}_{\text{ij}}\right)$$

As mentioned earlier, ${k}_{ij}$ is an adjustable parameter. Including this variable in the association explains the interactions between the two separate chains. The following definition has been provided for the relationship between the compressibility factor (Z) and $\tilde{a}^{res}$^28^:

26 $$Z = 1 + \eta \left( {\frac{{\partial \tilde{a}^{res} }}{\partial \eta }} \right)_{{T,x_{i} }} = 1 + Z^{hc} + Z^{disp}$$

The following methodology is used to establish the fugacity coefficient of component k (${\varphi }_{k}$)^28^:

27 $$ln\varphi_{k} = \tilde{a}^{res} + \left( {Z - 1} \right) + \left( {\frac{{\partial \tilde{a}^{res} }}{{\partial x_{k} }}} \right)_{{T,v,x_{j} \ne k}} - \sum \left[ {y_{i} \left( {\frac{{\partial \tilde{a}^{res} }}{{\partial x_{k} }}} \right)_{{T,v,x_{i} \ne j}} } \right]\, - \,\ln \,Z$$

## Results and discussion

### Sumatriptan solubility in SC-CO_2_

**Fig. 2:**
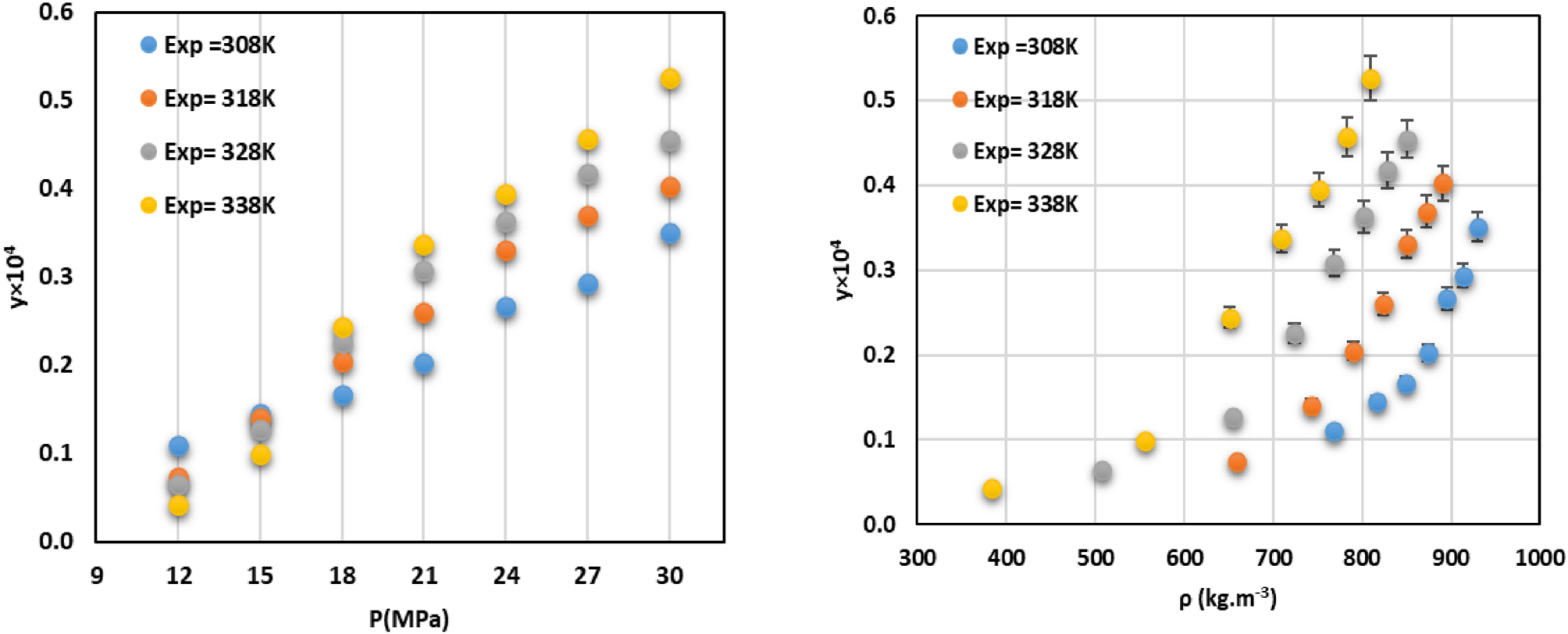
The experimental data verses pressure and density.

The mole fraction and solubility of Sumatriptan in SC-CO_2_ was meticulously measured using a static method across four isotherms: 308.2 K, 318.2 K, 328.2 K, and 338.2 K. For each isotherm, pressure was systematically varied from 10 to 30 MPa. The solubility system apparatus has been verified using *decitabine* solubility data (Table S1)^34^. The experimental conditions, along with the corresponding SC-CO_2_ densities, and the measured mole fraction solubilities of sumatriptan, are comprehensively presented in Table 3. The data represent the mean of three reproducible measurements, with the relative standard deviation consistently below 5%, affirming the high precision and reliability of the experimental data. The solubility of sumatriptan exhibited a clear and consistent trend; it increased significantly with increasing pressure at a fixed temperature. This phenomenon is primarily due to the direct relationship between pressure and the density of SC-CO_2_. As pressure increases, the density of the solvent rises, reducing the average intermolecular distance between CO_2_ molecules. The higher density means there are more solvent molecules per unit volume. This results in more favorable interactions between the drug and CO_2_ and enhances the solvating power of SC-CO_2_ for sumatriptan. For instance, at 308.2 K, the mole fraction increased from approximately 0.11 × 10^−4^ at 10 MPa to 0.351 × 10^−4^ at 30 MPa. Similar significant increases were observed across all isotherms. The influence of temperature on Sumatriptan solubility proved to be more complex due to the interplay of two opposing effects: the reduction of SC-CO_2_ density with enhancement of temperature at constant pressure, and the impact of the sublimation vapor pressure of Sumatriptan with increasing temperature. This interplay results in the well-known crossover phenomenon observed in supercritical fluid systems. The data revealed a crossover pressure for Sumatriptan in SC-CO_2_ occurring approximately between 12 and 15 MPa. At pressures below the crossover point, an increase in temperature at constant pressure led to a decrease in sumatriptan mole fraction. In this region, the reduction in SC-CO_2_ density (and thus its solvating power) with rising temperature was the dominant factor, overriding the effect of increased solute vapor pressure. In contrast, at pressures above the crossover point, an increase in temperature resulted in increased sumatriptan solubility. In this higher-pressure regime, the effect of Sumatriptan’s significantly increased sublimation vapor pressure with rising temperature became predominant. This outweighs the relatively smaller decrease in solvent density, leading to enhanced solubility^34–41^. This complex behavior is vividly illustrated in Fig. 2. The maximum observed mole fraction solubility for Sumatriptan was 0.526 × 10^−4^ at 30 MPa and 338.2 K, confirming the moderate potential of SC-CO_2_ as a solvent for Sumatriptan under appropriate conditions.

**Table 3:** Experimental mole fraction (y) of sumatriptan in supercritical carbon dioxide.

T (K)	P (MPa)	ρ_CO2_ (kg/m^3^)	y × 10^4^	Standard deviation × 10^6^	Solubility (g/L)
308.0	12	768.52	0.110	0.493	0.0531
	15	816.10	0.145	0.994	0.0760
	18	816.10	0.166	0.699	0.0914
	21	874.55	0.202	0.691	0.1153
	24	895.66	0.267	0.799	0.1565
	27	913.70	0.294	0.969	0.1763
	30	929.85	0.351	1.158	0.2144
318.0	12	659.80	0.074	1.502	0.0292
	15	743.83	0.141	0.590	0.0664
	18	790.29	0.205	0.859	0.1043
	21	823.80	0.261	0.988	0.1397
	24	850.15	0.331	0.999	0.1836
	27	872.14	0.369	1.950	0.2108
	30	890.90	0.402	1.995	0.2351
328.0	12	506.75	0.065	0.574	0.0190
	15	654.94	0.127	0.881	0.0517
	18	724.13	0.226	0.991	0.1044
	21	768.74	0.308	0.981	0.1530
	24	801.92	0.363	1.407	0.1894
	27	828.51	0.418	1.799	0.2262
	30	850.83	0.455	1.347	0.2536
338.0	12	384.17	0.043	0.364	0.0100
	15	555.33	0.099	0.682	0.0337
	18	651.28	0.244	0.898	0.1005
	21	709.59	0.338	1.348	0.1541
	24	751.27	0.394	1.728	0.1918
	27	783.19	0.457	2.982	0.2333
	300	809.68	0.526	1.507	0.2786

### Semi-empirical models

To effectively correlate and predict the data of sumatriptan in SC-CO_2_, four widely recognized density based models were employed. These models relate the solubility to the density and temperature of the CO_2_, without requiring complex pure properties or binary interaction parameters that are difficult to obtain experimentally. The performance of each model was evaluated using some statical criteria like AARD and R^2^.

1. Chrastil Model^19^: This model assumes a chemical association between solute and solvent molecules. The linearized form allows for the determination of an association number and an apparent enthalpy of dissolution. This model provided a reasonable fit to the data, with an AARD of 11.49% and R_adj_ of 0.985.
  29 $${\text{lny}}_{2}={a}_{0}+{a}_{1}ln{\rho }_{1}+\frac{{a}_{2}}{T}$$
2. Bartle et al. Model^20^: This model modifies the Chrastil equation by incorporating an additional term to account for the temperature dependency of the association number. It performed similarly to the Chrastil model, yielding an AARD of 13.58% and R_adj_ of 0.955.
  30 $$ln\left(\frac{{y}_{2}P}{{P}_{ref}}\right)={a}_{0}+\frac{{a}_{1}}{T}+{a}_{2}\left({\rho }_{1}-{\rho }_{ref}\right)$$
3. K-J Model^22^: This model, characterized by its flexible functional form, often provides superior fits for complex systems. For Sumatriptan, the K-J model demonstrated the best performance, achieving an AARD of 8.21% and R_adj_ of 0.991. This low AARD indicates that the K-J model is highly effective in describing the relationship between Sumatriptan solubility and the operational parameters of SC-CO_2_
  .
  31 $$ln{y}_{2}={a}_{0}+{a}_{1}{\rho }_{1}+\frac{{a}_{2}}{T}$$
4. MST Model^21^: The Modified Solubility Thermodynamics (MST) model, while conceptually robust, typically performs well for systems where strong solute–solvent interactions are present. For Sumatriptan, this model yielded an AARD of around 11.56% and R_adj_ of 0.975.
  32 $$Tln\left({y}_{2}P\right)={a}_{0}+{a}_{1}{\rho }_{1}+{a}_{2}T$$

**Fig. 3:**
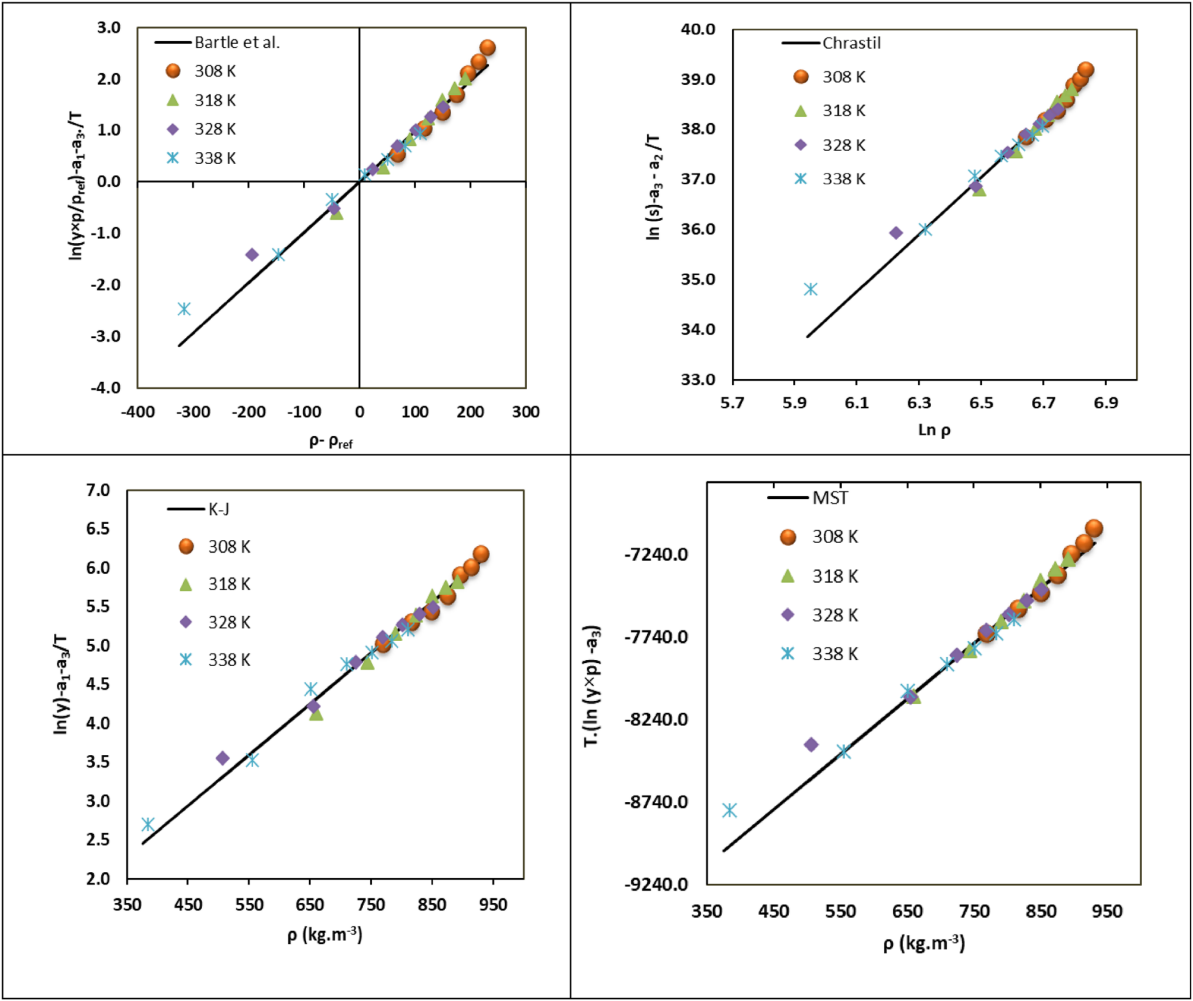
Density based models results.

The model shows a linear relationship across a wide temperature range except at densities below approximately half of the critical density of CO_2_ as a solvent. These linear trends allow for solubility extrapolation across temperatures and provide a means of verifying the consistency of solubility data. Researchers such as Shojaee et al.^42^; Tamura et al.^43^; Sabegh et al.^44^ have used this model to evaluate the consistency of experimental data by plotting $T(\text{ln}\left(y.P\right)-{a}_{3}$ against density (ρ), where consistent data should form a straight line, as shown in Fig. 3d.

In addition, as previously mentioned, Bartle et al. (1991) introduced a new equation to correlate the solubility data of solids in SC-CO_2_. There is a relationship between parameter ${a}_{1}$ and the enthalpy of sublimation of the solid solutes ${\Delta H}_{s}$:

$$\Delta H_{s} = - {\text{R}}a_{1}$$

Numerous researchers have successfully applied Bartle’s model in conjunction with Eq. 18. These researchers have found that the estimated sublimation enthalpies consistently align with the values reported in other studies (Yamini et al.^45,46^, Sodeifian et al.^47^, Rojas et al.^48^, Nasri^49^). The value of the sublimation enthalpy of sumatriptan (${\Delta H}_{s}$ = 59.83 kJ mol^−1^) is consistent with previous research^17,50–55^.

The correlation results for all density-based models are summarized in Table 4. This table present the AARD and R^2^ values for each model, along with their regressed parameters. The superior performance of the K-J model (lowest AARD of 8.21% and highest R^2^ of 0.991) is evident and further visualized in Fig. 3. This figure would show plots of experimental vs. correlated solubility for each model, highlighting the K-J model’s closer fit.

**Table 4:** The parameters of density-based models.

Model	${a}_{0}$	${a}_{1}$	${a}_{2}$	AARD%	R_adj_
Bartle et al	13.86	0.0097	− 7196.1	13.58	0.955
Chrastil	5.697	− 24.72	− 4927.6	11.49	0.985
K-J	− 0.81	0.0031	− 4813.3	08.21	0.991
MST	3.359	− 10,296.3	18.43	11.56	0.975

Furthermore, the Chrastil and Bartle models allowed for the estimation of apparent thermodynamic parameters associated with the dissolution process. Based on the regressed parameters (e.g., '${a}_{2}$' from Chrastil and Bartle), the apparent total heat of dissolution (${\Delta H}_{t}$) and the vaporization heat (${\Delta H}_{vap}$) for sumatriptan were estimated. The solvation heat (${\Delta H}_{sol}$), calculated as the difference between ${\Delta H}_{vap}$ and ${\Delta H}_{t}$, provides insight into the exothermic nature of the solute–solvent interaction. Specific values for sumatriptan are approximately: ${\Delta H}_{t}$ = 40.96 kJ mol^−1^, and ${\Delta H}_{vap}$ = 59.83 kJ mol^−1^, leading to an estimated ${\Delta H}_{sol}$ = − 18.87 kJ mol^−1^). These values confirm that the dissolution of sumatriptan in SC-CO_2_ is an endothermic process overall, driven by the strong entropic gain and favorable solvation interactions.

### Equations of state

For a more rigorous thermodynamic description of sumatriptan’s solubility in SC-CO_2_, three widely used EoSs were employed. These EoS models provide a fundamental framework for describing the phase behavior of fluid mixtures. To apply these models, pure component critical properties for sumatriptan were first estimated using group contribution methods, as they are not readily available experimentally for such complex molecules (See Table S2). Summarizes these estimated parameters:

1. Boiling Temperature (T_b_): Estimated at approximately 830.5 K using Boublia et al. method^56^.
2. Critical Temperature (T_c_): Estimated at approximately 1160.3 K using Boublia et al. method^56^.
3. Critical Pressure (P_c_): Estimated at approximately 3.39 MPa using Boublia et al. method^56^.
4. Acentric Factor (ω): Estimated at approximately 0.647 using Boublia et al. method^56^.
5. Molar Volume (V_m_): Estimated at approximately 305.2 cm^3^/mol using Cao et al. method^57^.
6. Sublimation Vapor Pressure (P_vap_): Calculated using the modified Clausius–Clapeyron equation, showing significant temperature dependence (e.g., at 308.2 K, Pvap ≈ 2.88 × 10^−8^ Pa; at 338.2 K, Pvap ≈ 3.45 × 10^−6^ Pa)^58^.

**Fig. 4:**
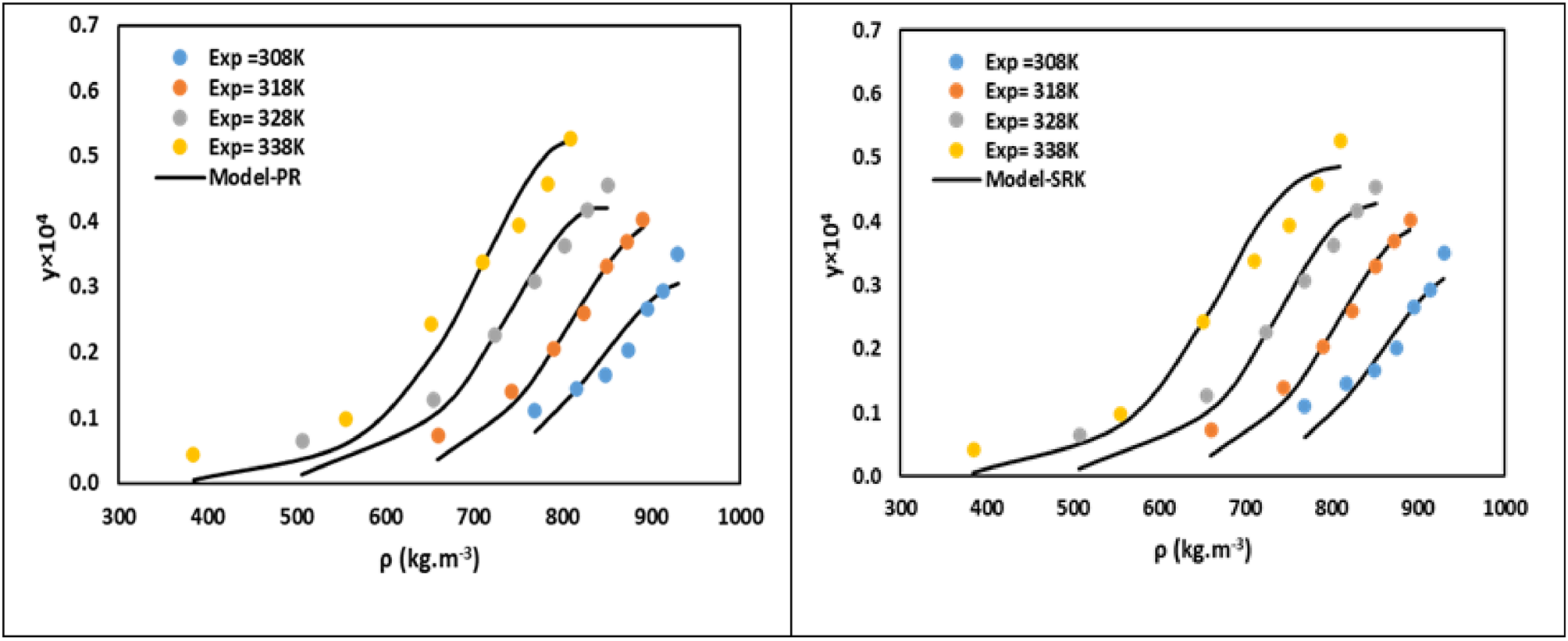
PR and SRK models outcome for mole fraction of sumatriptan.

To account for the non-ideal interactions between sumatriptan and CO_2_, mixing rules with two parameters (${k}_{ij}$, ${l}_{ij}$) were utilized. These parameters were calculated by regressing the experimental solubility for each isotherm. The correlation results for both EoS models are presented in Tables 5 and 6 including AARD and R_adj_ for PR-VdW2 and SRK-VdW2, along with the regressed ${\text{k}}_{\text{ij}}$ and ${\text{l}}_{\text{ij}}$ for each isotherm. The results show that the PR-VdW2 model demonstrated a slightly better overall performance, yielding an AARD of approximately 16.27% and an R_adj_ of 0.966. The SRK-VdW2 model also provided a reasonable fit, with an AARD of approximately 16.43% and an R_adj_ of 0.961. The performance of both EoS models is visually depicted in Fig. 4a, b. These figures would show parity plots or predicted vs. experimental mole fraction, demonstrating the fit of PR-VdW2 and SRK-VdW2.

**Table 5:** Outcome of modeling of sumatriptan solubility by PR.

Model	Parameter	T = 308 K	T = 318 K	T = 328 K	T = 338 K	Mean
PR-vdW2	k_ij_	− 0.084	− 0.107	− 0.192	− 0.223	
	l_ij_	− 0.448	− 0.507	− 0.732	− 0.812	
	AARD %	12.33	11.01	16.84	24.91	16.27
	R_adj_	0.969	0.979	0.963	0.956	0.966

**Table 6:** Outcome of modeling of sumatriptan solubility by SRK.

Model	Parameter	T = 308 K	T = 318 K	T = 328 K	T = 338 K	Mean
SRK-vdW2	k_ij_	− 0.0099	− 0.0622	− 0.1474	− 0.2561	
	l_ij_	− 0.2791	− 0.4081	-0.6343	− 0.9352	
	AARD %	13.32	12.19	18.13	22.11	16.43
	R_adj_	0.971	0.976	0.953	0.946	0.961

Furthermore, Tables 5 and 6 the presented that ${k}_{ij}$ *and* ${l}_{ij}$ parameters against temperature illustrates the temperature dependency of the parameters ${k}_{ij}$ *and* ${l}_{ij}$. Both parameters exhibited a negative linear correlation with temperature, meaning their values tended to decrease as temperature increased. This behavior is commonly observed in supercritical systems and reflects the changing nature of intermolecular forces and clustering phenomena with varying thermal energy. The linear approximation can be useful for predicting these parameters within the studied range.

### PC-SAFT equation of state

**Fig. 5:**
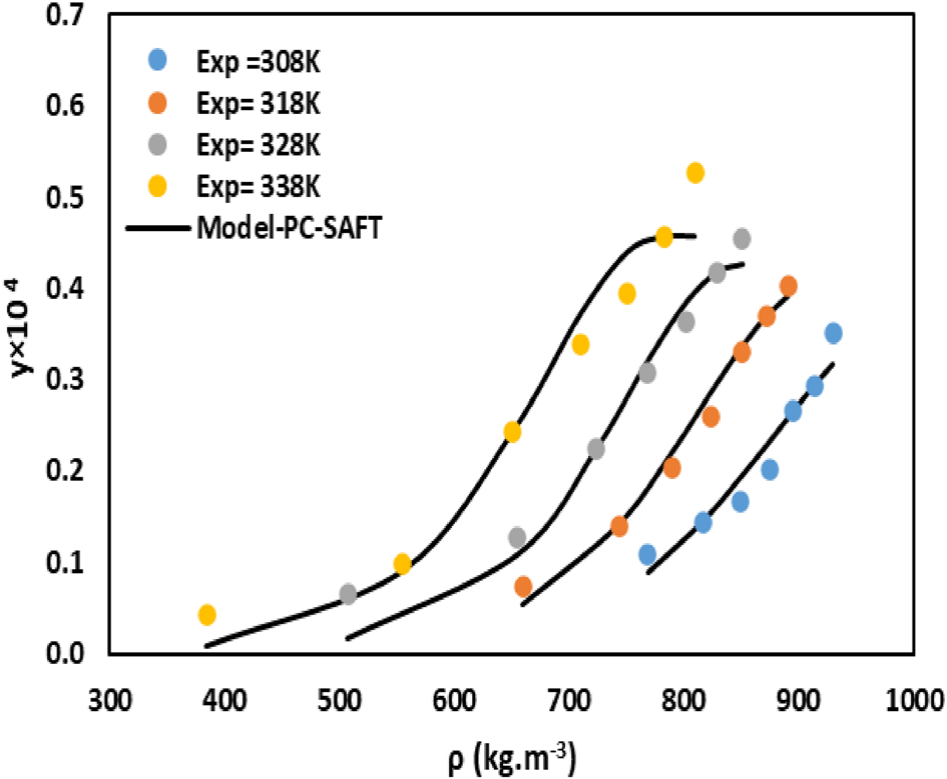
Experimental and predicted solubility at 308, 318, 328, and 338 K by PC-SAFT.

The PC-SAFT was utilized to model sumatriptan’s solubility in SC-CO_2_, with interaction parameters optimized using genetic algorithm. Table 7 provides temperature dependent parameters, and Fig. 5 compares experimental and predicted solubility at 308, 318, 328, and 338 K. The model achieved an overall AARD of 11.75% and an R_adj_ of 0.988, demonstrating high predictive accuracy, especially at elevated temperatures, consistent with literature findings. This precision underscores PC-SAFT’s effectiveness for supercritical solubility modeling. Several recent studies have advanced the modeling of pharmaceutical drug solubilities in CO_2_. For instance, Zhang et al.^59^ employed the PC-SAFT equation of state to model the solubility of ten anticancer drugs, such as capecitabine and docetaxel, at temperatures between 308 and 348 K and pressures ranging from 100 to 400 bar. Their molecular based model achieved superior accuracy (AARD below 10%) by optimizing three temperature independent parameters and considering association interactions. This model outperformed semi-empirical and cubic models. Similarly, Sodeifian et al.^60^ examined the solubility of gefitinib hydrochloride with sPC-SAFT and achieved a satisfactory balance of accuracy (an AARD of 10.18%) and computational efficiency. He et al.^61^ conducted a comparative study of PC-SAFT, empirical models, and machine learning approaches to predict the solubility of chlorothiazide and chloroquine. They found that PC-SAFT was robust, with absolute deviations below 5.25%. However, artificial neural networks provided the highest predictive accuracy. In addition, Hsu et al.^62^ evaluated the influence of cosolvents on drug solubility and found that PC-SAFT effectively models complex ternary systems involving topiramate and meclizine, achieving AARD below 10%. Overall, the application of EoS models provided a more fundamental, thermodynamically consistent framework for describing the complex phase equilibrium of sumatriptan in SC-CO_2_, offering valuable insights for process simulation and design.

**Table 7:** The results of PC-SAFT for solubility of sumatriptan.

Model	Parameter	T = 308 K	T = 318 K	T = 328 K	T = 338 K	Mean
PC-SAFT	k_ij_	− 0.128	− 0.161	− 0.179	− 0.201	
	AARD %	7.24	08.93	14.25	16.56	11.75
	*R*_*adj*_	0.993	0.990	0.986	0.981	0.988

## Conclusion

Enhancing the solubility and dissolution rate of active APIs through micronization or nanonization is a key strategy to improve therapeutic efficacy and minimize side effects. This comprehensive study successfully determined the solubility of sumatriptan in SC-CO_2_ across a broad range of industrially relevant temperatures (308.2–338.2 K) and pressures (10–30 MPa). The experimental mole fraction solubility, spanning from 0.43 × 10^−5^ to 0.526 × 10^−4^, demonstrated a clear dependency on solvent density and solute vapor pressure, characterized by a distinct crossover phenomenon. The experimental data were robustly correlated using four semiempirical models, with the K-J model proving to be the most accurate, achieving an AARD of 8.21%. This model offers a reliable and straightforward tool for predicting sumatriptan solubility in SC-CO_2_. Additionally, a rigorous thermodynamic analysis employing the Peng-Robinson and Soave–Redlich–Kwong with two parameter mixing rules provided further insights. The PC-SAFT model delivered a good correlation with an AARD of approximately 11.75%, and the determined binary interaction parameters showed a clear negative temperature dependency. The findings from this investigation are invaluable for advancing the understanding of sumatriptan’s phase behavior in SC-CO_2_. The validated experimental data and established predictive models are critical resources for the rational design, optimization, and scale up of green, supercritical fluid-based processes, such as particle size reduction, coprecipitation, or encapsulation, aimed at improving the pharmaceutical properties and manufacturing sustainability of sumatriptan. This research contributes significantly to the development of advanced pharmaceutical technologies for enhanced drug delivery.

## Supplementary Information

Supplementary Information.

## Data Availability

The datasets used and/or analysed during the current study available from the corresponding author on reasonable request.
