# Supplementary material for: Experimental and thermodynamic modeling of sumatriptan solubility in supercritical carbon dioxide for green pharmaceutical applications
Source: Sci Rep. 2025 Oct 21;15:36635. doi: 10.1038/s41598-025-20442-7 (PMC12540891; doi:10.1038/s41598-025-20442-7)
Supplement: Supplementary file 1 — Supplementary Information. [file 41598_2025_20442_MOESM1_ESM.docx]

**Experimental and Thermodynamic Modeling of Sumatriptan Solubility in Supercritical Carbon Dioxide for Green Pharmaceutical Applications**

Saud Bawazeer*

Department of Pharmaceutical Science, College of Pharmacy, Umm Al-Qura University, Makkah, Saudi Arabia,

Email:  [ssboazar@proton.me](mailto:%20ssboazar@proton.me)

***Table S1****: comparison solubility data of decitabine in this work and Pishnamazi et al.[1].*

| T | P=120 (bar) | P=160 (bar) | P=200 (bar) | P=240 (bar) | P=280 (bar) | P=320 (bar) | References |
| --- | --- | --- | --- | --- | --- | --- | --- |
| 308 K | 5.04×10⁻⁵ | 8.23×10⁻⁵ | 1.18×10⁻⁴ | 1.37×10⁻⁴ | 1.76×10⁻⁴ | 1.97×10⁻⁴ | Pishnamazi et al. |
| 308 K | 4.91×10⁻⁵ ±1.96×10⁻⁶ | 8.15×10⁻⁵ ±3.26×10⁻⁶ | 1.14×10⁻⁵ ±4.56×10⁻⁷ | 1.32×10⁻⁵ ±5.28×10⁻⁶ | 1.76×10⁻⁵ ±7.04×10⁻⁶ | 1.89×10⁻⁵ ±7.56×10⁻⁶ | This work |
| 318 K | 4.51×10⁻⁵ | 9.37×10⁻⁵ | 1.55×10⁻⁴ | 1.87×10⁻⁴ | 2.40×10⁻⁴ | 2.69×10⁻⁴ | Pishnamazi et al. |
| 318 K | 4.66×10⁻⁵ ±1.86×10⁻⁶ | 9.30×10⁻⁵ ±3.72×10⁻⁶ | 1.61×10⁻⁵ ±6.44×10⁻⁶ | 1.85×10⁻⁵ ±7.40×10⁻⁶ | 2.39×10⁻⁵ ±9.56×10⁻⁶ | 2.55×10⁻⁵ ±1.02×10⁻⁵ | This work |
| 328 K | 3.69×10⁻⁵ | 9.11×10⁻⁵ | 1.77×10⁻⁴ | 2.82×10⁻⁴ | 3.42×10⁻⁴ | 4.27×10⁻⁴ | Pishnamazi et al. |
| 328 K | 3.66×10⁻⁵ ±1.46×10⁻⁶ | 9.21×10⁻⁵ ±3.68×10⁻⁶ | 1.78×10⁻⁴ ±7.12×10⁻⁵ | 2.93×10⁻⁴ ±1.17×10⁻⁴ | 3.50×10⁻⁴ ±1.40×10⁻⁴ | 4.12×10⁻⁴ ±1.74×10⁻⁴ | This work |
| 338 K | 2.84×10⁻⁵ | 7.79×10^–5^ | 2.05×10^–4^ | 3.71×10^–4^ | 4.90×10^–4^ | 7.06×10^–4^ | Pishnamazi et al. |
| 338 K | 2.75×10⁻⁵ ±1.10×10⁻⁶ | 7.66×10⁻⁵ ±3.07×10⁻⁶ | 2.03×10⁻⁴ ±8.12×10⁻⁵ | 3.61×10⁻⁴ ±1.44×10⁻⁴ | 4.86×10⁻⁴ ±1.94×10⁻⁴ | 7.19×10^4^  ±1.74×10⁻^5^ | This work |

**Table S2.** Critical properties calculator.

| **Groups** | **Ring / No ring** | ***∆TbMi*** | ***∆TcMi*** | ***∆PcMi*** | ***∆VcMi*** | ***∆Mi*** | **Comp 1** |
| --- | --- | --- | --- | --- | --- | --- | --- |
|  |  |  |  |  |  |  |  |
|  |  |  |  |  |  |  |  |
| **-CH3** | **No ring** | 23.58 | 0.0275 | 0.3031 | 66.81 | 15.035 | 3 |
| **-CH2-** | **No ring** | 22.88 | 0.0159 | 0.2165 | 57.11 | 14.027 | 3 |
| **>NH** | **No ring** | 50.17 | 0.0119 | 0.0322 | 78.96 | 15.015 | 1 |
| **>N-** | **No ring** | 11.74 | -0.0028 | 0.0304 | 26.7 | 14.007 | 1 |
| **=CH-** | **Ring** | 26.73 | 0.0114 | 0.1693 | 42.55 | 13.019 | 4 |
| **=C<** | **Ring** | 31.01 | 0.0051 | 0.0955 | 31.28 | 12.011 | 4 |
| **>NH** | **Ring** | 52.82 | 0.0244 | 0.0724 | 27.61 | 15.015 | 1 |
| **-SO2** | **No ring** | 147.24 | -0.0563 | -0.0606 | 112.19 | 64.065 | 1 |
|  |  |  |  |  |  |  |  |
|  |  |  |  |  | **Mw (g/mol)** | | **295.408** |
|  |  |  |  |  | **Tb (K)** | | **830.510** |
|  |  |  |  |  | **Tc (K)** | | **1160.367** |
|  |  |  |  |  | **Pc (bar)** | | **33.952** |
|  |  |  |  |  | **Vc (mL/mol)** | | **919.290** |
|  |  |  |  |  | **ω** | | **0.6477** |

1 Pishnamazi, M. Experimental and thermodynamic modeling decitabine anti cancer drug solubility in supercritical carbon dioxide. *Sci. Rep.* **11**, doi:10.1038/s41598-020-80399-7 (2021).
